# FDG PET/CT as the Decisive Modality in Distinguishing Clear Cell Sarcoma from Melanoma When Immunohistochemistry Overlaps: A Case Report

**DOI:** 10.1055/s-0046-1819651

**Published:** 2026-03-25

**Authors:** Efrah Ahmed Ibrahim, Eray Alper

**Affiliations:** 1 Department of Nuclear Medicine, Bursa Uludağ University Hospital, Bursa, Türkiye; 2 Department of Nuclear Medicine, Bursa Uludağ University Faculty of Medicine, Bursa, Türkiye

**Keywords:** clear cell sarcoma, malignant melanoma, FDG PET/CT, differential diagnosis, SOX10, melanoma of unknown primary

## Abstract

Clear cell sarcoma (CCS) and malignant melanoma share overlapping immunohistochemical profiles, particularly SOX10 and HMB45 positivity, making histopathological differentiation challenging. Distinguishing between these entities is clinically essential due to their differing prognoses and therapeutic approaches. This case highlights how fluorine-18 fluorodeoxyglucose positron emission tomography/computed tomography ( ^18^ F-FDG PET/CT) contributed decisively to establishing the diagnosis of CCS by following a pathologist-recommended diagnostic algorithm. A 70-year-old male presented with a progressively enlarging left scapular mass. Tru-cut biopsy revealed a malignant neoplasm positive for SOX10 and HMB45, with immunohistochemistry insufficient to differentiate CCS from metastatic melanoma. The pathology report recommended a search for a primary cutaneous melanoma, indicating that in the absence of such a lesion, the diagnosis should favor CCS. Whole-body ^18^ F-FDG PET/CT demonstrated extensive metastatic disease involving the lungs, left adrenal gland, T11 vertebra, scapula, and a large presacral mass. Critically, no hypermetabolic cutaneous, mucosal, or nodal lesions suggestive of a primary melanoma were identified. Comprehensive dermatological evaluation was also negative for suspicious melanocytic lesions. Based on the diagnostic algorithm, histopathology, plus exclusion of a primary melanoma, the PET/CT findings supported the diagnosis of metastatic CCS. This case demonstrates that ^18^ F-FDG PET/CT can serve as a diagnostic tool when histopathology alone is inconclusive. The absence of a primary melanoma on whole-body ^18^ F-FDG PET/CT and clinical examination was the decisive factor favoring CCS over melanoma of unknown primary. This case underscores the limitations of MRI, which often describes CCS as a well-defined, benign-appearing mass, potentially delaying accurate diagnosis.

## Introduction

Clear cell sarcoma (CCS) was first described by Enzinger in 1965 as a distinct entity arising from tendons and aponeuroses. 1 Often called “melanoma of soft parts,” CCS shares a common embryological origin with malignant melanoma; both arise from neural crest cells and can produce melanin. 2 This biological similarity translates into overlapping immunohistochemical profiles, with both tumors typically expressing melanocytic markers including S100, HMB45, SOX10, and Melan-A. 3 4 This creates a real diagnostic problem when you are looking at a biopsy specimen without a clear clinical context.

The distinction between these two entities matters a lot. Malignant melanoma, particularly in the metastatic setting, has seen dramatic improvements in outcomes with the advent of immune checkpoint inhibitors (pembrolizumab, nivolumab) and targeted therapies for BRAF-mutant disease, with response rates of 40 to 70% and median survival now exceeding 2 to 3 years. 5 6 CCS, unfortunately, remains largely resistant to conventional chemotherapy and shows limited responses to newer systemic agents, with a median survival in metastatic disease around 12 to 13 months. 7 8

When a patient presents with metastatic disease from a melanocytic tumor without an obvious primary site, the diagnosis of melanoma of unknown primary (MUP) is often considered. MUP accounts for approximately 2 to 6% of all melanoma cases. 9 The workup typically involves a thorough search for an occult or regressed primary lesion, including complete skin examination and imaging. Fluorine-18 fluorodeoxyglucose positron emission tomography/computed tomography ( ^18^ F-FDG PET/CT) has become the standard imaging modality for staging melanoma and detecting occult disease. 10

Conventional imaging modalities like magnetic resonance imaging (MRI) and CT have limitations in characterizing CCS. Multiple studies have shown that CCS often has a deceptively benign appearance on MRI, presenting as well-defined, homogeneous, lobulated masses with smooth margins. 11 12 In fact, up to 52% of CCS cases show slightly increased signal intensity on T1-weighted MRI images compared with muscle, a feature related to melanin content, but the overall morphology frequently mimics benign soft tissue tumors. 12 This benign-looking appearance on anatomical imaging can delay diagnosis or lead to mischaracterization of the lesion.

We present a case where the combination of histopathology, PET/CT findings, and clinical examination led to the diagnosis of CCS in a patient with widespread metastatic disease.

## Case Report

Written informed consent was obtained from the patient for the publication of this case report and any accompanying images.

### Clinical Presentation

A 70-year-old male patient was referred to our nuclear medicine department for ^18^ F-FDG PET/CT staging following the discovery of a large left scapular mass. The patient had undergone Tru-Cut biopsy of the scapular lesion, which revealed malignant tumor infiltration.

### Histopathology

The pathology report described a tumor with a solid-organoid growth pattern, separated by fibrous septa. The tumor cells showed plasmacytoid morphology with eccentric nuclei, eosinophilic cytoplasm, and occasional intranuclear inclusions. The differential diagnosis was broad, including carcinoma metastasis, plasma cell myeloma, PEComa, malignant melanoma, epithelioid sarcoma, and CCS.

Immunohistochemical staining revealed:

- Positive: HMB45 (focal), SOX10, SATB2.
- Negative: CK, CK7, CK20, GATA-3, TTF-1, Napsin A, CDX2, PAX8, EMA, SALL4, Hepar, CD99, BCOR, CD138, Lambda, Kappa, LCA.

Histochemical staining showed:

- Silver stain: Rich reticular framework.
- PAS: Negative.
- D-PAS: Negative.

The pathologist concluded that the HMB45 and SOX10 positivity indicated a melanocytic tumor but could not definitively distinguish between malignant melanoma and CCS. The pathology report specifically stated:

“It is recommended that the patient be examined for cutaneous malignant melanoma metastasis, including the anal region and nail beds, and if skin malignant melanoma is not found, the malignant melanoma of soft tissue should be evaluated as 'Clear Cell Sarcoma'.”

### PET/CT Imaging

**Fig. 1:**
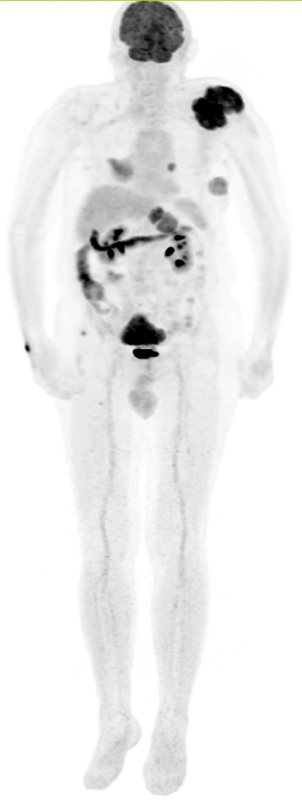
Whole-body fluorine-18 fluorodeoxyglucose ( ^18^ F-FDG) positron emission tomography (PET) maximum intensity projection (MIP) image demonstrating multiple hypermetabolic lesions throughout the body, including bilateral lung nodules, left adrenal gland mass, T11 vertebral lesion, left scapular mass, large presacral mass, and multiple soft tissue lesions.

The patient underwent whole-body ^18^ F-FDG PET/CT imaging from the skull base to mid-thigh. The study was performed 60 minutes after intravenous administration of 296 MBq (8 mCi) of ^18^ F-FDG following a 6-hour fast. Blood glucose level was 98 mg/dL at the time of tracer injection. The maximum intensity projection image ( Fig. 1 ) demonstrates multiple hypermetabolic lesions distributed throughout the body. These include bilateral pulmonary nodules, a hypermetabolic mass in the left adrenal gland, a lesion involving the T11 vertebral body, a left scapular lesion, a large presacral mass, and multiple hypermetabolic soft-tissue lesions. A detailed summary of the PET/CT findings is provided in Table 1 .

**Table 1:** Summary of ^18^ F-FDG PET/CT findings

Anatomical site	Lesion size (mm)	SUV _*max*_	Description
**Primary lesion**			
Left scapula	109 × 105 × 84	13.1	Fig. 2 ; large destructive bone lesion infiltrating soft tissues and muscles (biopsied site)
**Distant metastases**			
Right lower lobe lung	52 × 39	6.2	Fig. 3 ; lobulated mass with pleural involvement and bronchial obliteration
Left lower lobe lung	24 × 20	5.7	Fig. 3 ; nodule in postero-basal segment
Left adrenal gland	49 × 43	7.7	Fig. 4 ; large hypermetabolic mass
T11 vertebra	Not applicable	8.2	Fig. 5 ; lytic destructive lesion involving the left half of the body and posterior elements
Presacral region	117 × 95 × 73	15.6	Fig. 6 ; large lobulated mass (highest metabolic activity)
Left chest wall	48 × 43	5.3	Soft tissue lesions
Right gluteal muscle	Not applicable	4.5	Focal hypermetabolic focus
Right anterior thigh	Subcentimetric	2.6	Subcutaneous nodule
**Other findings**			
Right thyroid lobe	22 × 17	2.7	Hypodense nodule with peripheral calcification
Right perirenal region	16 × 11	1.9	Small nodule

Abbreviations: CT, computed tomography; ^18^ F-FDG, fluorine-18 fluorodeoxyglucose; PET, positron emission tomography.

Note: All lesions demonstrated increased ^18^ F-FDG uptake relative to background activity. SUV _*max*_ values are reported for all measurable lesions; “Not applicable” indicates lesions without reliable size measurement due to partial volume.

**Fig. 2:**
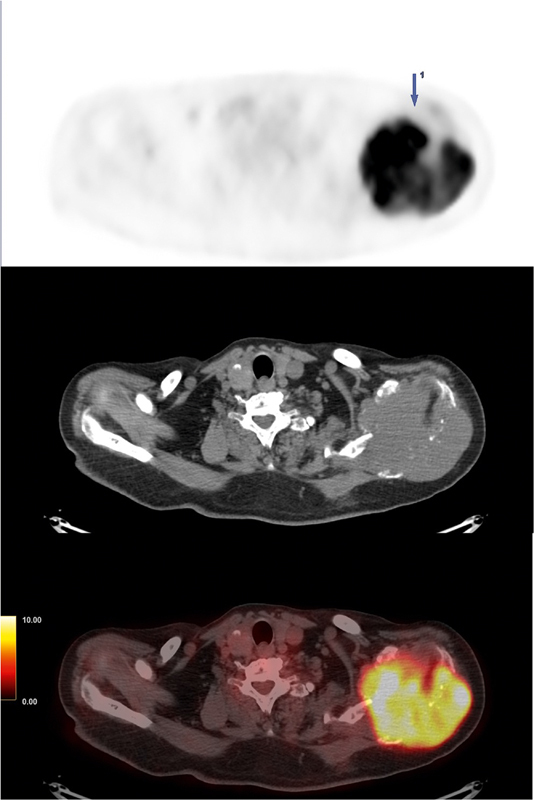
Composite ^18^ F-FDG PET/CT images of the left scapula lesion. From top to bottom: PET, CT, and fused PET/CT images. The images demonstrate a large, destructive bone lesion (109 × 105 × 84 mm) with intense FDG uptake (SUV _*max*_ 13.1) and infiltration into the surrounding soft tissues and muscles. This was the site of the diagnostic biopsy that revealed HMB45 and SOX10-positive tumor. CT, computed tomography; ^18^ F-FDG, fluorine-18 fluorodeoxyglucose; PET, positron emission tomography.

**Fig. 3:**
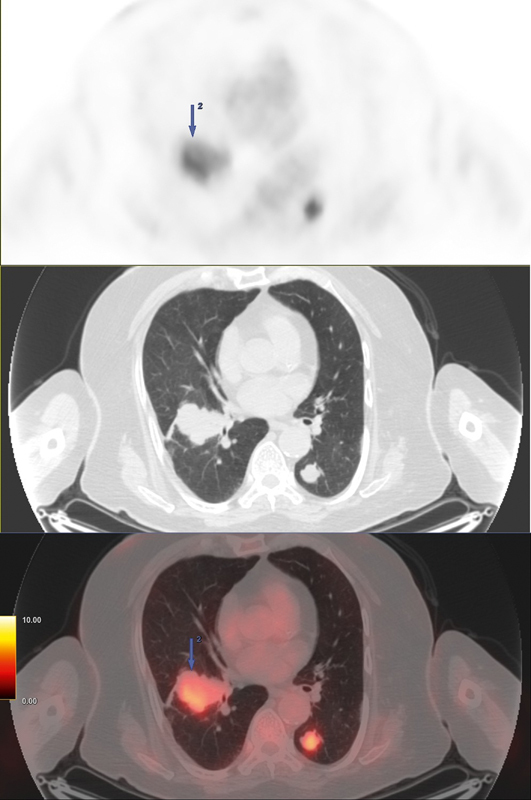
Composite ^18^ F-FDG PET/CT images of bilateral lung metastases. From top to bottom: PET, CT, and fused PET/CT images. The images show multiple metastatic lung lesions. The largest is a lobulated mass in the right lower lobe (52 × 39 mm, SUV _*max*_ 6.2) with pleural involvement and bronchial obliteration. Another significant nodule is seen in the left lower lobe (24 × 20 mm, SUV _*max*_ 5.7). CT, computed tomography; ^18^ F-FDG, fluorine-18 fluorodeoxyglucose; PET, positron emission tomography.

**Fig. 4:**
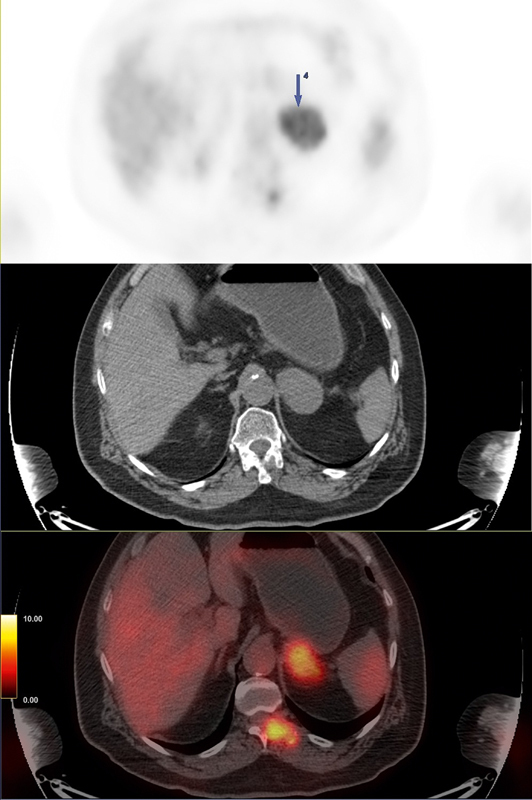
Composite ^18^ F-FDG PET/CT images of the left adrenal gland metastasis. From top to bottom: PET, CT, and fused PET/CT images. The images show a large, hypermetabolic lesion in the left adrenal gland (49 × 43 mm, SUV _*max*_ 7.7), consistent with adrenal metastasis. CT, computed tomography; ^18^ F-FDG, fluorine-18 fluorodeoxyglucose; PET, positron emission tomography.

**Fig. 5:**
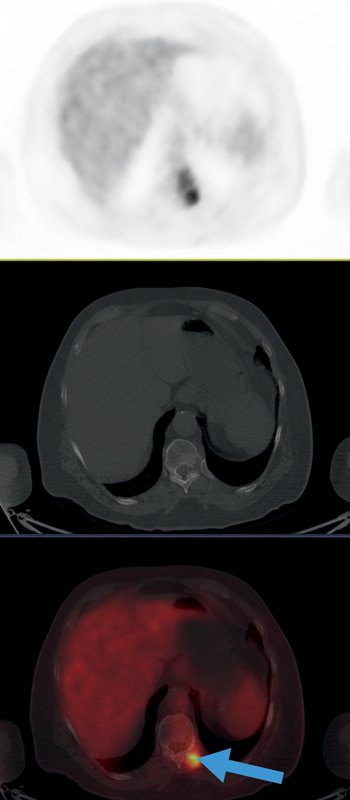
Composite ^18^ F-FDG PET/CT images of the T11 vertebral metastasis. From top to bottom: PET, CT, and fused PET/CT images. The images demonstrate a lytic, destructive lesion involving the left half of the T11 vertebral body and posterior elements, with intense FDG uptake (SUV _*max*_ 8.2). The blue arrow on the fused image (bottom panel) points to the hypermetabolic vertebral lesion. CT, computed tomography; ^18^ F-FDG, fluorine-18 fluorodeoxyglucose; PET, positron emission tomography.

**Fig. 6:**
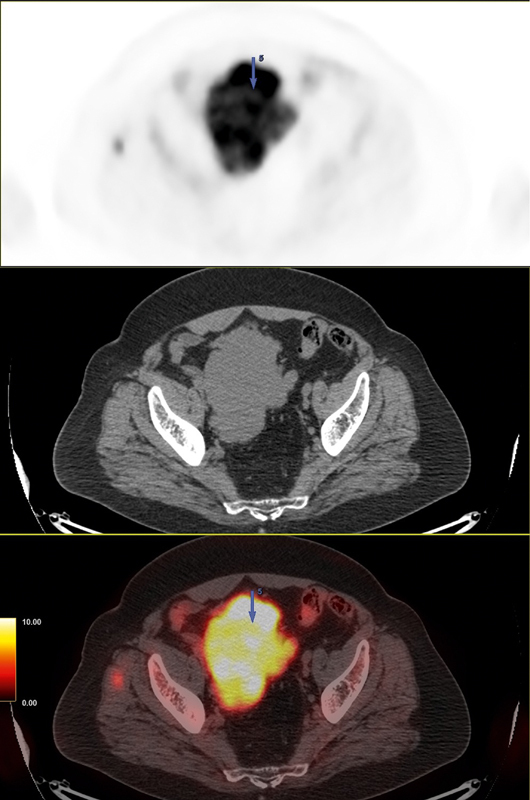
Composite ^18^ F-FDG PET/CT images of the presacral mass. From top to bottom: PET, CT, and fused PET/CT images. The images reveal a large, lobulated presacral soft tissue mass (117 × 95 × 73 mm) with markedly increased FDG uptake (SUV _*max*_ 15.6), representing the highest metabolic activity in the entire body. CT, computed tomography; ^18^ F-FDG, fluorine-18 fluorodeoxyglucose; PET, positron emission tomography.

Crucially, the whole-body PET/CT scan did not identify any hypermetabolic skin or mucosal lesion that could represent a primary melanoma.

### Clinical Examination

Following the PET/CT findings, the patient underwent a thorough dermatological examination, including the anal region and nail beds, as recommended by the pathologist. A detailed dermatological evaluation revealed only benign melanocytic and keratinocytic lesions (regular nevi, seborrheic keratoses, and lentigo simplex) without any clinically suspicious or atypical pigmented lesions. No evidence of cutaneous, acral, or mucosal melanoma was detected, thereby excluding a primary melanoma source.

### Diagnosis

Based on the pathologist's diagnostic algorithm, the absence of a primary cutaneous melanoma on both comprehensive PET/CT imaging and dermatological examination, combined with the presence of a large soft tissue mass in the scapula, the diagnosis of CCS with widespread metastases was established.

## Clinical Course and Follow-Up

Following the ^18^ F-FDG PET/CT examination, the patient was referred for a comprehensive dermatological evaluation as recommended in the pathology report. Full-body skin examination, including scalp, acral regions, nail beds, genital, and anal mucosa, revealed no primary cutaneous or mucosal melanoma.

FDG-avid lesions identified on PET/CT guided subsequent targeted biopsies from multiple metastatic sites, including the rectosigmoid mass and soft-tissue lesions. All biopsy specimens demonstrated malignant melanocytic tumor infiltration with immunohistochemical features concordant with the initially sampled scapular lesion, confirming metastatic disease. No evidence of a primary gastrointestinal or cutaneous melanoma was identified.

The case was reviewed in a multidisciplinary oncology council, where the integrated histopathological, imaging, and clinical findings favored the diagnosis of metastatic CCS. Combination immune checkpoint inhibitor therapy (ipilimumab and nivolumab) was initiated under an initial working diagnosis of metastatic malignant melanoma before final diagnostic integration. Palliative radiotherapy was delivered to symptomatic osseous lesions. Given the advanced disease stage, treatment was focused on symptom control.

## Discussion

This case illustrates an important diagnostic challenge in oncology, distinguishing between two melanocytic malignancies that share immunohistochemical markers but have fundamentally different clinical behaviors and treatment approaches. The pathologist faced a tumor that was clearly malignant and clearly melanocytic (HMB45 + , SOX10 + ), but these markers alone could not separate melanoma from CCS. This is not surprising since both tumors originate from neural crest cells and express the same melanocytic differentiation markers. 3 4

### The Diagnostic Algorithm

The pathologist's approach was logical and evidence-based. When confronted with a melanocytic tumor of uncertain type, the first step is to search for a primary cutaneous melanoma. If a skin primary is found, the diagnosis is metastatic melanoma. If no skin primary is found despite thorough examination, and the tumor arises from soft tissue (especially near tendons), then CCS becomes the more likely diagnosis. 13 CCS should be considered in any melanocytic tumor arising from deep soft tissue without an identifiable cutaneous primary. This diagnostic algorithm was explicitly stated in our pathology report.

### Role of PET/CT in the Diagnostic Workup

PET/CT is highly sensitive for detecting melanoma, with reported sensitivities of 80 to 90% for lesions larger than 5 to 10 mm. 10 14 The whole-body scan provided a comprehensive survey covering all common sites for primary melanoma. The absence of any suspicious cutaneous or mucosal hypermetabolic lesion provided strong evidence against the diagnosis of metastatic melanoma.

### The Unusual Metastatic Pattern

One aspect of this case that deserves discussion is the extent of metastatic disease. CCS typically presents as a localized tumor in the distal lower extremity. While it can metastasize, the pattern we saw here with bilateral lung involvement, adrenal metastasis, vertebral destruction, and a massive pelvic mass is more commonly associated with melanoma than CCS. 15 16 In fact, this widespread visceral involvement initially made us consider melanoma as the more likely diagnosis.

However, CCS can behave aggressively. While uncommon, cases of CCS with extensive metastases have been reported. 17 The key point is that the metastatic pattern alone is not diagnostic; it is the combination of findings that matters. Yes, the distribution looked more like melanoma, but the absence of a primary cutaneous lesion and the presence of a large soft tissue mass in the scapula (which could serve as the primary tumor) tipped the balance toward CCS.

## Limitations of Conventional Imaging

It is worth noting that conventional anatomical imaging has significant limitations in characterizing CCS. A large multicenter MRI study of 21 CCS cases found that these tumors often have a “benign-looking appearance” on MRI, with well-defined margins, homogeneous signal, and lobulated contours. 12 Up to 52% of CCS cases show slightly increased signal intensity on T1-weighted images compared with muscle, which is related to melanin content. 12 However, this mild hyperintensity on T1 MRI does not necessarily correlate with aggressiveness; it simply reflects the melanocytic nature of the tumor. Overall, the morphology frequently mimics benign soft tissue tumors like schwannomas or myxomas. 11 12

This benign appearance on MRI can be misleading. In our case, if we had relied solely on the morphological characteristics of the scapular mass on CT (which showed a large destructive lesion but with relatively well-defined borders), we might not have appreciated the aggressive nature of the disease. The metabolic information from PET showing intense FDG uptake and widespread metastases provided crucial information about disease biology that anatomical imaging alone could not offer.

### The Importance of Molecular Testing

The definitive test to distinguish CCS from melanoma is molecular analysis. CCS characteristically harbors the t(12;22)(q13;q12) translocation resulting in EWSR1-ATF1 gene fusion (or less commonly EWSR1-CREB1), which can be detected by fluorescence in situ hybridization or reverse transcription polymerase chain reaction. 18 19 Melanoma, in contrast, often shows BRAF mutations (∼50% of cases) or NRAS mutations, but lacks the EWSR1 fusion. 20

The absence of this test in our case represents a limitation due to institutional constraints. In such situations, the integration of histopathology, imaging findings, and clinical examination becomes essential for diagnosis. Our case demonstrates that this integrated approach, guided by the pathologist's recommendations, can lead to a reasonable diagnostic conclusion even without molecular confirmation. Molecular confirmation using EWSR1-ATF1 or EWSR1-CREB1 fusion analysis was not performed due to institutional resource constraints at the time of evaluation. In this real-world context, diagnostic integration of histopathology, whole-body PET/CT, FDG-guided biopsies, and clinical exclusion of melanoma was essential to reach a clinically actionable diagnosis.

## Clinical Implications

The distinction between CCS and melanoma has major therapeutic implications. For metastatic melanoma, we now have multiple effective treatment options. Immune checkpoint inhibitors (pembrolizumab, nivolumab, ipilimumab) have revolutionized melanoma treatment, with response rates of 40 to 45% for anti-PD-1 monotherapy and even higher for combination regimens. 5 For BRAF-mutant melanoma, targeted therapy with BRAF/MEK inhibitors (dabrafenib/trametinib, vemurafenib/cobimetinib) produces rapid responses in 60 to 70% of patients. 6 Five-year survival rates for metastatic melanoma have improved dramatically, now reaching 30 to 40% with modern therapies. 21

For CCS, the picture is much bleaker. The tumor is notoriously resistant to conventional chemotherapy. 7 Newer agents like sunitinib have shown some activity (response rate around 30% in small series), and there are case reports of responses to immunotherapy, but overall, systemic treatment options remain limited and largely ineffective. 8 22 Median survival for metastatic CCS is only 12 to 13 months. 7 The mainstay of treatment remains surgical resection for localized disease, but for a patient like ours with widespread metastases, treatment options are very limited. In our case, the negative finding (no primary melanoma) was the key piece of information that allowed us to follow the pathologist's diagnostic algorithm and arrive at the diagnosis of CCS.

### Broader Implications for PET/CT Utilization

We are accustomed to thinking of PET/CT as a tool for detecting disease, but the absence of disease in expected locations can be equally informative. Other examples of this principle include:

- Excluding a primary tumor in cancer of unknown primary syndrome. 23
- Ruling out inflammatory/infectious etiologies in fever of unknown origin. 24
- Confirming complete metabolic response after therapy. 25

## Limitations

Our case has some limitations. First, we do not have molecular confirmation with *EWSR1-ATF1* fusion testing, which would be definitive. Second, the patient's clinical follow-up and response to treatment would provide additional validation of the diagnosis. Finally, the unusual extent of metastatic disease in this case makes it somewhat atypical for CCS, though well-documented cases of aggressive metastatic CCS do exist in the literature.

## Conclusion

We present a case where ^18^ F-FDG PET/CT played a crucial diagnostic role in distinguishing CCS from malignant melanoma, two melanocytic tumors with overlapping immunohistochemical profiles. The key contribution of PET/CT was not the detection of metastases, but rather the absence of a primary cutaneous or mucosal lesion.

This case demonstrates that PET/CT functions not only as a staging tool but also as a diagnostic instrument when integrated with histopathology and clinical findings. The case also highlights the limitations of conventional anatomical imaging modalities like MRI, which often show CCS as benign-appearing, well-defined lesions that can be misleading. In the era of personalized medicine, where accurate diagnosis determines treatment selection, ^18^ F-FDG PET/CT provides valuable information beyond simple disease staging. This case underscores how PET/CT can provide diagnostic clarification in cases where histopathology alone is inconclusive.
